# The challenges of implementing advanced access for residents in family medicine in Quebec. Do promising strategies exist?

**DOI:** 10.1080/10872981.2018.1438719

**Published:** 2018-02-21

**Authors:** Sabina Abou Malham, Nassera Touati, Lara Maillet, Mylaine Breton

**Affiliations:** ^a^ Department of Community Health Sciences, Université de Sherbrooke, Charles Lemoyne Hospital Research Centre, Longueuil, Québec, Canada; ^b^ École nationale d’administration publique, Montréal, Québec, Canada; ^c^ Institut Universitaire de première ligne en santé et services sociaux, Centre integre universitaire de sante et services sociaux de l'Estrie, Sherbrooke, Québec, Canada

**Keywords:** Advanced access, challenges, implementation, promising strategies, teaching clinical settings

## Abstract

**Background**: The advanced access (AA) model is a highly recommended innovation to improve timely access to primary healthcare. Despite that many studies have shown positive impacts for healthcare professionals, and for patients, implementing this model in clinics with a teaching mission for family medicine residents poses specific challenges.

**Objective:** To identify these challenges within these clinics, as well as potential strategies to address them.

**Design**: The authors adopted a qualitative multiple case study design, collected data in 2016 using semi-structured interviews (*N* = 40) with healthcare professionals and clerical staff in four family medicine units in Quebec, and performed a thematic analysis. They validated results through a discussion workshop, involving many family physicians and residents practicing in different regions

**Results**: Five challenges emerged from the data: 1) choosing, organizing residents’ patient; 2) managing and balancing residents’ appointment schedules; 3) balancing timely access with relational continuity; 4) understanding the AA model; 5) establishing collaborative practices with other health professionals. Several promising strategies were suggested to address these challenges, including clearly defining residents’ patient panels; adopting a team-based care approach; incorporating the model into academic curriculum and clinical training; proactive and ongoing education of health professionals, residents, and patients; involving residents in the change process and in adjustment strategies.

**Conclusions**: To meet the challenges of implementing AA, decision-makers should consider exposing residents to AA during academic training and clinical internships, involving them in team work on arrival, engaging them as key actors in the implementation and in intra- and inter-professional collaborative models.

## Introduction

Advanced access (AA) is a highly recommended innovation for improving timely access to primary healthcare services [1,2]. It is one of the main components of the patient-centered medical home model, which is characterized by patient-centered practice of medicine to improve the performance of primary healthcare, including quality and access to care [3,4]. AA is based on five guiding principles (Table 1): 1) balancing supply and demand (assessing the actual patient demand for appointments by physician per day, weighted by the patients’ status and balancing the supply—e.g., number of appointments offered—to meet the potential needs of the clientele); 2) reducing appointment backlog (eliminating previously waiting lists through many strategies, such as adding resources or increasing the supply of visits during a period of time, and putting in place a communication strategy to educate patients about the new AA model); 3) reviewing the appointment system (planning physicians’ schedules over 2–4 weeks and smoothing out the demand for visits in order to offer same-day appointments for acute and urgent cases); 4) integrating inter-professional practice (developing or enhancing inter-professional practice between physicians and other healthcare providers through optimizing the roles of each professional involved in the process including nurses and matching patient needs to skills of adequate health care provider, to respond to their needs in a timely manner); 5) developing contingency plans during periods of absence and high demand (planning for seasonal increases in demand and developing coverage plans for replacing medical staff or other healthcare providers during vacation and sick leave) [1].

**Table 1.** The key principles of advanced access, adapted from [1,16].

Key principles of Advanced Access	Definition
1. *Balance supply and demand*	To assess and understand, on the one hand, the actual patient demand for appointments per physician per day, weighted by the patients’ status and, on the other hand, the supply (e.g., number of appointments offered), in order to achieve the right balance between the two, matching demand with supply. Strategies to decrease demand for visits (e.g., max pack, extending visit intervals) or to increase supply (e.g., redesigning doctor scheduling systems) are used.
2. *Reduce the backlog of previously scheduled appointments*	To eliminate previously scheduled appointments (wait list) through many strategies, such as adding resources or increasing the supply of visits during a period of time. A communication strategy must also be put in place to inform and educate patients about the new advanced access model.
3. *Review the appointment system*	To plan physicians’ schedules over a short term (2–4 weeks) and smooth out the demand for visits in order to offer same-day appointments for acute and urgent cases.
4. *Integrate inter-professional practice*	To develop or enhance inter-professional practice between physicians and other healthcare providers (e.g., nurses). Professional roles need to be optimized and tasks need to be clarified to respond to patient needs in a timely manner.
5. *Develop contingency plans*	To plan for seasonal increases in demand and to develop coverage plans for replacing medical staff or other healthcare providers during vacation and sick leave. Many strategies are applied, such as increasing the number of slots prior to leave and after returning to duty, hiring temporary providers, and distributing and matching staffing competencies to demand. Integrating collaborative and interdisciplinary practice facilitates planning for periods of absence.

AA ensures patients obtain an appointment at the date and time that suits them, within a period of time appropriate to their condition, while responding in an optimal way to their expected needs [1,5].

Among the organizational settings experimenting with implementation of this model in Canada, the USA, and internationally, are family medicine clinics with a teaching mission. Such clinics have great potential for spreading innovation among future healthcare professionals [6], as they provide an opportunity for involving residents at an early stage of training in optimizing access to care, by reorganizing their medical practice according to the AA model and adopting it in their future practice [4,7].

However, international research has shown that clinics, medical teams, and residents in particular face sizeable challenges when implementing AA [8,9]. Despite the significance of the specific challenges faced by residents [10], only a few studies have considered them.

These studies have documented the lack of availability and variation in family physician supply [7,10] resulting from the fragmentation of training from one place to another, and the obligation for residents to undertake training in different environments (e.g., emergency, hospitalization), which threatens continuity of care during their training in clinics [7,9,11,12]. Moreover, results concerning impacts on relational continuity are inconsistent. While some studies point to a relational discontinuity that undermines the continuity principle of the AA model [6,11,13], others show that involving residents in the implementation process generates positive results such as improving patient enrolment [14], continuity of care [7,14], and efficiency within the team [14].

Many countries attempting to achieve the goals of improved access and patient-centered care are increasingly implementing AA, as a component of the patient-centered medical model. However, evaluation is still under development. In addition, the challenges faced by residents remain under-investigated [7]. A recent experiment by Groulx et al. [6] in deploying AA for residents in a rural medical primary care practice revealed challenges related not only to general organizational practices in the unit (lack of nursing resources, lack of patient education) but also specifically to residents (e.g., lack of availability, internship obligations). Adjustments have been suggested in certain areas (e.g., appointment duration, proportion of same-day appointments, and patient volumes) to help residents practice AA in accordance with the skills required by the College of Canadian Family Physicians (CanMEDS-Family Medicine).

Given the inherent contextual characteristics of teaching units [8,14,15] (e.g., fluctuations in resident’s clinical schedules, part-time work of supervising physicians), and the scarcity of data on resident challenges, current gaps in knowledge must be filled in order to improve accessibility of care. The **objective** of this article is to identify the challenges associated with implementing AA for residents in family medicine, as well as potential strategies to overcome these challenges.

## Methods

In Quebec, AA implementation is a growing priority for the Federation of General Practitioners of Quebec (FMOQ) and the Ministry of Health and Social Services (MSSS), to address the long-standing problem of accessing services [16]. Many Family Medicine Units (FMUs), primary healthcare organizations with a teaching mission, have already switched to the AA model, and many more will soon follow. A qualitative comprehensive approach was adopted to reflect the personal experiences of actors concerning issues faced in daily practice [17–19]. We conducted a multiple case study and selected four FMUs to understand implementation challenges and potential strategies to be promoted in a teaching environment. Units with ‘early adopter’ profiles were selected based on two criteria: 1) an implementation period greater than 1 year; 2) diversity of socio-demographic environments (rural and urban FMUs) (see Table 2).

**Table 2.** Characteristics of the selected family medicine units.

	FMU 1	FMU2	FMU3	FMU4
Setting	Urban	Urban	Urban	Rural
IUHSSC	A	B	C	D
**Team composition**				
Family physicians	33	20	13	15
Residents 1st, 2nd year (R1-R2)	25	24	13	14
Advanced practice nurse	2	1	1	1
Registered nurse	4	4	1	2
Clerical staff	4	4	2	4
**Registered patients**	11,000	10,000	< 6,000	6,700
**Patient population served**	All types, ages (Pediatric, pregnant women, young families, elderly, vulnerable patients, etc.)	All types, ages	All types, ages	All types, ages

FMU: family medicine unit; IUHSSC: Integrated University Health and Social Services Center; R1: first year of residency; R2: Second year of residency

Participants were recruited through the FMUs’ medical directors, who were contacted to identify key stakeholders involved in the change process and using the AA model. A purposeful and diversified sampling strategy targeted five categories, composed of health professionals (residents [R], family physicians [FP] including the unit medical directors [MD], nurse practitioners [NP], clinical nurses [N]) and clerical staff [CS]. An interview guide was used, which covered perceptions of the challenges, modalities for adapting the AA model, and suggestions on organizing the AA model for residents.

Semi-structured interviews (*N* = 40), lasting from 40 to 60 min, were conducted between May 2015 and February 2016. They were carried out face-to-face or by telephone when constrained by distance (distance ≥ 150 km). Data saturation was attained by the tenth interview within each FMU.

Ethical approval was obtained from the Center for Health and Social Services—Sherbrooke University Institute of Geriatrics. The informed consent of each participant was obtained at the beginning of the interview.

Following an open coding approach, all transcribed interviews were coded using QDA Miner version 4.0 (Provalis Research, 2011). The first author has conducted (SAM) coding and reverse-coding using an iterative process, and one co-author (MB) reviewed a random sample. All themes emerging from the respondents’ verbatim statements were validated with all co-authors.

An inductive approach was adopted to conduct an intra- and inter-case analysis that highlighted mainly commonalities (similarities) and some differences regarding the challenges of implementing AA at the different sites under study. A coding grid developed during the analysis was used for this purpose. In this article, transversal analysis is the core focus of the results.

Credibility was ensured through a reflective triangulation strategy [20], which consisted in returning the final interpretations to current AA users in a teaching unit for validation. To this end, an academic workshop was held in September 2016 at the annual meeting of a university family medicine department with numerous supervising physicians and residents (*N* = 20) practicing in six different FMUs across several regions. The three-hour workshop provide the opportunity to: 1) gather written and oral data on users’ experiences implementing this model in a teaching unit in general and among residents in particular; and 2) verify the results and validate promising strategies for facilitating implementation of the model by residents. A dynamic interactive approach was adopted involving group work and plenary discussion. A researcher observed and audio-recorded the discussions, with the consent of participants, and took summary notes. Sharing results with stakeholders corroborated our findings and enhanced our understanding of the challenges and possible solutions.

## Results

Results are discussed in terms of the study’s two objectives.

### Objective 1: identify the perceived challenges of implementing AA for residents in FMUs

Although the level of implementation of AA principles varied across the four study practices, all FMUs reported four similar challenges: 1) choosing and managing the residents’ patient panel; 2) concurrently ensuring a balance between timely access and relational continuity of care 3) managing and balancing the residents’ appointment schedules; and 4) getting residents to understand and appropriate the model. A fifth challenge, establishing collaborative practice, was reported at two FMUs (cases 2 and 3) that had failed to implement some of the AA principles, as compared to others (cases 1, 4) (Table 3)

**Table 3.** Challenges faced by residents and level of implementation of advanced access principles across four family medicine units.

		Sites—Family medicine units								
		1	2	3	4		1	2	3	4
		Challenges of implementing advanced access					Level of implementation of the key principles of advanced access			
				
Choosing and organizing the patient population		√	√	√	√	P1. Balancing supply and demand	√	x	N/A	√
Ensuring balance between access and continuity of care		√	√	√	√	P2. Reducing the backlog	√	No waiting list	New FMU using advanced access	√
Managing the residents’ schedules		√	√	√	√	P3. Reviewing the appointment system	√	√	√	√
						P4. Integrating/amplifying inter-professional collaborative practice	√	x	x	√
Understanding and appropriating the model		√	√	√	√					
Putting in place collaborative practice			√	√		P5. Developing contingency plans	√	x	√	√

FMU: Family Medicine Units; N/A: Not applicable;

**Table 4.** Challenges and promising strategies to facilitate the implementation of advanced access for residents.

Specific challenges related to organizing residents’ practice according to the advanced access model	Strategies/possible solutions identified by the participants	Illustrative quotes
1. Choosing and managing the residents’ patient population● Assignment of patients by type● Patient panel size● Distribution of cases (e.g, complex cases) *according to residents’ variable pedagogical needs* and the level of their competencies	– Clear definition of residents’ patient populations● Adaptation of the caseload according to the learning objectives and the level of residents’ skills (R1-R2).● Preparation of a patient list for each resident at the beginning of the training● Regular re-evaluation of the number of patients in residents’ caseloads, considering patient demands, the diversity of health problems of the assigned patients, and the complexity of follow-up.	*‘I think that, for residents, it’s important that they have their own pool of patients and that they see their patients. For them as well, I think that for their practice, since they’re starting out, it’s important that at the beginning, during the first year, they know, “I’m going to see these patients, to prepare myself.” You know, it’s important that there shouldn’t always be patients added on at the last minute. For the sake of preparation. Maybe for preparation at the beginning; but I think it’s also to learn to manage their clientele that way, for their future practice, it’s important to know already how they can optimize their practice.’ (FP2-FMU2)*
2. Concurrently ensuring balance between access and continuity of care● Variable and limited availability (residents assigned to various clinical settings)● Impact on residents’ ability to provide rapid access and to meet patients’ needs in a timely manner● Breakdown (disruption) of relational continuity, a lack of conformity with the skills required by their pedagogical curriculum.3. Managing residents’ appointments schedules● Difficulty of planning, standardizing schedules; difficulty of organizing schedules to increase accessibility to patients while avoiding empty time slots● Difficulty of managing follow-up time when assigning patients with complex health problems	– Putting in place new collaborative models/practices (Ensuring relational continuity with the team members)● Twinning (pairing up) residents● Joint follow-up of a group of patients by the residents and the clinical nurse or the nurse practitioner● Follow-up of a group of patients by a small team (or a sub-team) (physicians, residents, nurses, clerical staff)– Address residents’ absences by using unfilled appointments of other residents to meet patients’ needs	*‘For adapted access […] to maybe eventually have more collaboration with the nurses, maybe for follow-up of chronic diseases, to not have to see them as often, to have more room for active patients, of course that could eventually help. I don’t see currently how it could be done, unless there was new personnel, but for sure if they could see, let’s say, our diabetics for every other visit, or our chronic patients every other visit, that would also help relieve a part of our work.’ (R2.2-FMU2)* *‘Of course, if we were the same four residents with the same nurse and we were following the same pool of 200 patients, that would be good. That would be very good. That way, the nurse could do some triage of what’s urgent, what is not urgent, then dispatch to the residents. That would be really wonderful.’ (R2.1-FMU2)* *‘I know there are places where they have a group of doctors with a nurse. For now, we’re not yet set up that way. We’d like to see it happen one day.’ (N-FMU2)* *‘So, yes, it means there has to be a way to think about this, especially when there is a lot of treatment happening, to restrict, to share letters. Also, sometimes the appointment can be with someone else; it could be the nurse, it could be the pharmacist, depending on the need.’ (FP4-FMU3)* *‘It’s clear that when they [the residents] are not there and that there is a need for follow-up, in that case they are obliged to refer to someone else.’ (NP2–FMU1)* *‘We have residents who can only come two, three times in a month […] Yes. Exactly [transferring to other residents]. We ask them whether it can wait until the resident’s return; if it can’t, well, then we rely on our algorithm and we go ahead and set up the appointment under advanced access.’ (CS-FMU3)* *‘In fact, residents are a population that, how should I say, they’re difficult physicians to manage, because they are only there two years, and on top of that, during their two years, they spend half the time outside of the clinic, then there are periods, but they come every week, then there’s two months that they spend in training outside, [..] when they’re in another training they come a half-day a week […], so then when they get here, let’s say they have two and half days of office time, so when they come one day a week, a half-day a week, they have too many patients, then when they come back the next month, they don’t have enough, so what we do is we balance it out. When people call for an appointment and there’s no space with the physician or the other resident has gone on his outside training, well it will go to a resident who has room. If his office is supposed to have 5 appointments and there are 3, well we’ll add 2 in the days before. Basically, as it should be in fact, he will compensate for the residents who are outside or rural areas, but when he is off on rural training, the other residents will see his patients, so we end up having more balanced schedules for them.’ (MD-FMU1)*
4. Understanding and appropriating the advanced access model	– Preparing and training residents, all health professionals, and the clerical staff● Including a course in the academic curriculum● Clinical education as soon as residents enter residency (e. g., workshop)● Standardizing advanced access learning concepts for supervisors– Educating also patients	*‘Just for that, I think that everyone should do it, so it’s good that we get training in that as soon as we begin residency training. Except right now it’s new, so there’s no formal training; but by doing it this way, we risk not being able to operate in advanced access once we’re done with our training.’ (R2-1-FMU3)* *‘But there were never that many courses on how to organize, let’s say, a clinical practice with advanced access… But it couldn’t hurt to also include it at some point somewhere along the way in our training.’ (R2.2-FMU2)* *‘I think that training in that area is very much lacking. Yes, from the first time I took training with the FMOQ, I had doubts about universities, I felt that it should really be taught from the first year of residency […] There could very well be arrangements that are also adapted to student’s time schedules, etc. That can be figured out. I think that if the role models, the bosses, practice it, then students will learn it through osmosis.’ (FP2-FMU4)* *‘Well, the way of doing things is a little different; we don’t see patients once a year just for an annual check-up, we have to do our examination every time they show up, split it up across each visit they make. It would be good, to have a few tips on how to organize our files to make sure we don’t miss anything, because now we don’t do everything every time, we have to do a little all the time. So, a little bit on the structure, how to be organized, how to plan out our appointments, in the longer term I think that would be valuable.’ (R2-1-FMU3)*
	– Adopting a collective approach to change● Engaging/involving residents in regular staff follow-up meetings, co-constructing work tools and adjustment strategies of advanced access● Engaging physicians and team members in the whole change process	*‘And then, to not hesitate, in fact, for FMUs, residents also have to be involved in the transformation, because for them it’s exposure to management, it’s one of the competencies they need to develop during the family medicine training process. I found it worthwhile to have residents join in the meetings. I thought it was good training, and they’re not exposed to that kind of situation […] For our part, we were doing it during lunch hours; of course residents, they have a lot of training, and they have a lot to learn, but still I found it worthwhile including them in the process to launch all of that.’ (FP2-FMU4)* *‘But I think it’s good to involve resident physicians and professionals-in-training in this way of doing things, so that they can already be familiar with this care method and be able to apply it later on in their practice.’ (NP1-FMU4)* *‘Well, I would say, it’s to train everyone together, to agree on how to proceed before starting.’ (FP4-FMU3)* *‘No, the equation is not that simple. No. It means that our first challenge is to make sure we all switch over to advanced access, that our residents function in advanced access, that our patients understand what’s going on, and that our staff is onboard as well.’ (FP1-FMU2)* *‘It’s really about training everyone beforehand, so you can agree on your model together. Then, after that, after – let’s say – six months of implementation, or, not even, three months, have another sit-down. But the thing is, here, no follow-up was done, no one was trained. I think we have to do training, do a follow-up at two months, at three months, at six months, ok, it’s working or not working, all sit down together. Then the whole team has to understand how it works and agree on it.’ (FP4-FMU3)*
5. Establishing collaborative practice	– Ensure that resources are available● Provide sufficient financial resources to increase the number of nurses, among other things.	

#### Choosing and managing residents’ patient panels

Across the sites, participants had quite similar perceptions overall. The method and criteria for assigning patients to a new cohort of residents, meeting both their learning needs and the clients’ demand for services, represented important challenges. Several supervisors reported the challenge of ensuring a balanced distribution of patients with complex health issues (e.g., multi-morbidities) among different residents, while adapting the type of patients to residents’ year of residency and to their heterogeneous educational needs.

“There is going to be an adjustment challenge. It will involve reorienting the patients, yes, but then we also want it to be balanced for the residents to learn. We want a variety of pathologies, so that each resident can follow a few diabetics, a few COPDs [chronic obstructive pulmonary diseases], […] Yes, that’s it. So, it has to be balanced” (MD-FMU2)

Providing access to services in a timely manner requires sharing complex and severe cases among the various residents, which remains a challenge.

“But of course we try to balance things so that one resident won’t have all the severe cases. I think the follow-up for more severe cases is somewhat evenly distributed among all the residents” (FP1-FMU1)

Some stressed the difficulty of preparing the patient caseload for the incoming first-year residents to practice the AA model.

“For residents who have just arrived, it’s not so feasible during the first year, because after all they have to be set up with a list of patients. On the other hand, I would say that, from about nine months in, we can get them started with advanced access.” (CS1-FMU4)

Another challenge reported by several participants was choosing the type of patients to be followed by residents (patients enrolled with residents, or other physicians’ and residents’ patients, etc.), while responding promptly to the demand for services. Some physicians (FMU 2) raised questions around this issue, namely, how to operate in an AA model that responds optimally to residents’ learning needs and level of competence.

“The question is, what the result is. Do we assign them a patient who has come as a walk-in and who isn’t their patient? Or do we assign more patients who are their patients and who need to be seen quickly.” (FP1-FMU2)

Others (FMU 1) have tried to diversify the clientele, avoiding empty appointment slots by providing quick appointments to other patients.

“I think that, at the beginning, we hadn’t set up fast appointments, then just in terms of unit operations, they weren’t seeing enough patients that way. That’s why I think that keeping slots that are really open, not just for their patients but to everyone, I think that’s a good idea for FMUs” (FP2-FMU1)

#### Concurrently ensuring a balance between timely access and relational continuity of care

A significant challenge reported by most participants in the four FMUs was the residents’ ability to provide timely access to patients and to meet their needs at all times, given their limited availability at the FMU. The main causes underlying the lack of availability, as identified by participants, were the fragmentation of training from one setting to another, the extensive mobilization of residents, and the diversification of clinical settings imposed on residents (e.g., in regions, hospitalization, and geriatrics).

“So, already that creates a problem in FMUs, because doctors don’t have much office availability, it’s the same thing for residents, who change over, or are at the hospital for the week, […] at a transitional functional rehabilitation unit, or at a geriatric unit and, in any case, if patients try to see them, well, they can be absent for 2 weeks with all the specialized activities that they have in Quebec City. They are often absent, and their presence is irregular. So, there are access problems.” (FP2-FMU2)

Access problems due to lack of availability often led to frustration among residents and discontent among patients.

“But they [residents] also experience frustration due to not being at the clinic often. It’s more difficult to work in advanced access when you’re not around that much. You have to be there to make it work properly.” (FP1-FMU3)

According to several participants, this not only disrupted the relational continuity with patients, but also led to deviation from the skills required by their pedagogical curriculum.

“Maintaining relational continuity is always difficult for residents and then because [….] they’re not always at the FMU, they leave for two months to remote areas, or three months on training, so basically they’re not always there either. For AA, I was saying that it was great [for patients] to see their own doctors for 48 hours, for two weeks, it was their doctor, which allowed a more long-term follow-up, but in the case of residents, if they’ve gone off to a rural area, the patient has again to see another resident again, I think it’s difficult.” (FP1-FMU1)

#### Managing and balancing residents’ appointment schedules

A particular challenge in residency programs is the varying availability of appointments from 1 week to another, due to the multi-site training required by the programs. The participants reported that residents’ variable schedules made it particularly challenging to manage, plan, and especially standardize their appointment schedules (e.g., duration of appointments, percentage of open slots, number of follow-ups per day). Organizing schedules to increase patient access, while avoiding empty slots and minimizing their negative consequences, was a particular challenge. For example, filling residents’ empty time slots with patients, whom they are not used to following, can sometimes lead to difficulty in managing the follow-up time for complex cases (e.g., patients with several pathologies and a heavy list of drugs) and consequently a greater delay for the patients booked later in the resident’s daily schedule.

“It’s maybe about managing time, because, well, we have a schedule with allocated time slots. For example, a resident is assigned an additional patient in his office. Well, the case may be more complex than he expected, so he may run over time, so the other patients are going to be delayed […] It could be harder for the residents because sometimes they will have patients with whom they’re not familiar. When they are in their office, they get prepared, they know their patients, they do everything, ‘my plan with this patient’ […]. which means it can be difficult for them.” (NP-FMU1)

In these teaching settings, another challenge reported is organizing and managing the residents’ schedules to match supervising physicians’ schedules, so that the latter can assume their teaching and clinical supervision responsibilities.

“It is about the residents’ highly variable schedules. More or less everyone’s in clinic, so it’s really about setting up the residents’ schedules and knowing that they do have available time slots. Residents also have to be paired with a physician who is available to supervise them. So, it’s mostly about schedule management […]. Implementation challenges, yes, yes, that’s it. In my opinion, it’s mainly on the organizational side.” (R1-FMU1)

#### Understanding and appropriating the advanced access model

A challenge unanimously reported by residents in all four FMUs was operating in AA when they had not been prepared nor exposed to the model during their academic training.

“I really mainly learned about that once I got to my residency training. During my clerkship, we did many specialty courses, so it was not really conveyed as a principle. It was really when I arrived in the family medicine residency that I heard about it. So, in any case, I personally had not been exposed to that at all.” (R2-FMU4)

Participants also pointed out the lack of supervision during residency internships in family medicine and the lack of adequate training strategies to understand the principles of AA and its operational modalities. The data revealed that most residents had received superficial and very limited information related only to the appointment review principle.

“Yes, well, you heard a little about it during the clerkship, but no, no one had really explained to me how it worked or how to set it up.” (R1-FMU1)

However, this challenge appeared to have been diminished at one FMU (case 4), where reflective learning exercises had been adopted to facilitate appropriation of the model.

“But also the bosses who had had training, well, in any case, I think they might have had more meetings than we did, so they were able to tell us, ‘Such-and-such a patient, you could put into advanced access,’ or they would make us think it over, telling us, ‘This patient here, do you think he should be in advanced access, or do you think he should be seen again in two months?’ […]. So, it was often pretty much like that, the bosses would challenge us a lot to see whether we, as residents, do we put our patients in adapted access or not, and for what reasons.” (R2-FMU4)

#### Establishing collaborative practice

Some participants pointed out that the challenge of implementing a joint follow-up model (e.g., nursing-residents) arose in two FMUs due to the lack of availability of financial and human resources (nurses) (FMU 3) and the lack of involvement in the implementation process (FMU 2).

An unfavorable organizational context in terms of financial and human resources, among other factors, can influence the implementation of AA for residents. Collaborative practice, in terms of interdisciplinary follow-up to optimize care, is a characteristic of a favorable context.

“Of course if we were the same four residents with the same nurse and we were following the same pool of 200 patients, which would be good. That would be very good. That way, the nurse could do some triage of what’s urgent, what is not urgent, then dispatch to the residents. That would be really wonderful. I feel that for the time being that’s somewhat wishful thinking, because… The fact is, it would require additional nursing resources.” (R2-1-FMU2)

### Objective 2: identify potentially promising strategies to facilitate the implementation of advanced access for residents

Although implementing AA for residents is challenging, solutions seem to exist to facilitate it in an educational setting (Table 4). They focus mainly on:

1. Identifying and revising the residents’ patient panels to balance demand for care with residents’ learning needs. This incorporates adapting the caseload according to the learning objectives and preparing a patient list for each resident that matches the level of residents’ skills at the beginning of the training. This step will be followed by a regular re-evaluation of the number of patients in residents’ caseloads throughout the training period, considering on one hand the patient demands, the diversity of health problems of the assigned patients, and on the other hand the trainees’ needs, their learning obligations and the complexity of follow-up.
2. Establishing new collaborative models: A variety of models can be implemented such as
  - Pairing up residents or developing resident care teams, each consisting of 2 or 4 residents (with different rotation schedules), who assume a collective responsibility towards a group of patients and ensure provision continuity of care.
  - Implementing group practice models such as *a joint follow-up of a* group of patients by the residents and the clinical nurse or the nurse practitioner, who will work in partnership and carry out coordinated follow-up visits for a diverse clientele (e.g., pregnant, patients with chronic illness); *or a small team* composed of multiple health care professionals (physicians, residents with different levels, nurses, clerical staff) who will work collaboratively and follow-up a group of patients in a way that any provider within this sub-team would be able to respond to their needs.
3. Preparing and training health professionals and residents on the various key principles of AA so they can integrate it into their practice: such as integrating a course in the curriculum related to the AA model that contributes to initiating them to different practice models during their academic program. Also, upon entering their residency training, they can be trained through attending workshops and also through exposing them to many cases seen under this practice redesign and providing them the appropriate supervision using quality improvements tools. Moreover, it would be essential to standardize AA learning concepts for supervisors to enable delivery of a unified lesson for residents, to clearly convey its key principles and bring the whole supervising members to work in the same direction.
4. Educating patients, using many methods (e.g., reminder cards, verbal explanation), should be performed on a regular basis to change their habits in using primary care services.
5. Involving residents and the whole team in a collective approach to change, within the limits of their availability and their academic obligations: Essential steps would be engaging residents in regular staff follow-up meetings, and involving them in co-constructing work tools and in adjustment strategies of AA. It would be also useful to develop key performance indicators relevant to the roles of residents and all health care professionals in achieving practice quality and efficiency.

## Discussion

Our findings shed much-needed light on a subject that has not been thoroughly investigated thus far. Research in this area has focused mainly on the challenges faced in a teaching context by the team in general. Few studies have addressed the challenges faced specifically by residents and how to overcome them [6]. Although some authors have acknowledged one of the main challenge raised in our study—the balance between accessibility and continuity in a residential unit [9,13]—they have not drawn a comprehensive picture of the implementation challenges. Potential solutions to this complex problem (e.g., reorganizing teamwork to mitigate the discontinuity due to residents’ frequent absences and variable schedules) remain elusive [9] or, at best, are still being tested [7]. By giving the different team members a chance to speak, we have highlighted not only the implementation challenges faced by residents but also the possible strategies to circumvent them, thus providing a global perspective. The perceived challenges are numerous and concern choosing and organizing the residents’ patient population, balancing accessibility and relational continuity of care, managing and balancing residents’ appointment schedules, understanding and appropriating the model, and establishing collaborative practice. They are also influenced by organizational challenges (e.g., lack of financial or professional resources, or leadership), as reported in a separate article [21] and in other research studies conducted abroad [1,22] that should not be overlooked.

Our results are in line with those reported by the Accessibility Committee of the Department of Family Medicine and Emergency Medicine at the University of Sherbrooke, which highlighted the same challenges that may compromise development of the key competencies required in the ‘CanMEDS-Family Medicine Competency Framework’ (College of Family Physicians, 2015) related to family medicine roles: management (patient and time management, supply and demand); collaboration (sharing of activities with other healthcare professionals); and, erudition (commitment to reflective learning, transfer of knowledge). Consequently, educational directors should pay particular attention to these findings to attain educational goals and prepare residents to provide high-quality, safe, patient-centered care.

Our results suggest concrete and proactive strategies to address these challenges and to facilitate implementation success. The strategies need to be implemented at an early stage in order to anticipate, minimize, or overcome the challenges.

Training residents, the entire health and clerical staff as well as patients; assigning residents an active role within a collective approach to change; and optimizing team work by establishing new collaborative models with residents were all put forward as promising strategies by key informants.

These strategies emerging from the field echo the findings of existing studies. For example, some authors have focused not only on resident involvement in implementing AA but also on strategies for improving and adapting the model through regular follow-up meetings throughout the entire change process [6,7]. Such strategies help to strengthen the commitment and motivation of all team members, and especially residents, throughout the practice redesign process [7] (e.g., to a patient-centered family medicine model), empowering them and contributing to the emergence of resident champions who can guide other residents in developing strategies for improving the quality of services provided to patients [23]. With respect to residents’ patients, Groulx et al. [6] also recommended determining a patient panel size that facilitates practicing the AA model in accordance with residents’ skill levels in the Canadian CAND program.

Some authors have also recommended integrating AA in resident training, without, however, focusing on the modalities of pedagogical learning required to achieve this [6,8]. Regarding collaboration, the literature focuses on new models in which a team-based care approach [15] is adopted to improve accessibility and promote organizational or team continuity, by instituting small teams—‘teamlets’—in charge of a panel of patients [24,25]. Practicing in interdisciplinary teams to improve management of residents’ patient panels was among the recommendations made by Wileand [26] to improve continuity of care.

Optimizing the role of other professionals through joint monitoring with nurses has also been raised [1,16] as a way to improve access to family physicians. However, with respect to residents, little has been written on developing contingency plans to alleviate the difficulty of providing rapid access to patients during residents’ absence from the clinic [6], or on evaluating their effects. A recent study by Butler et al. [12] showed that implementing a new ‘Resident care team’ model (e.g., twinning as part of a team of six to seven residents, mainly in charge of a group of patients, nurse and a trainee tutor) in an internal medicine clinic led to a significant improvement in informational, management, and relational continuity of care (88.9% versus 41.9%; *p* ˂0.0001). The potential of such a model in the context of AA remains to be explored.

Our results, which draw on several international scientific papers that emphasize the need to adapt this model for family medicine residents [14] as well as on the future direction of the Family Medicine Center model for all family medicine training networks [4] and professional organizations (College of Family Physicians of Canada) [27] in Canada, represent a real contribution to this field. They should serve to guide other primary healthcare settings with a teaching mission, enabling better prediction of implementation challenges and strategies for involving residents in AA adaptation, to improve accessibility and resident availability. They will also help in recommending strategies for supporting academic and clinical teams.

### Limitations

This qualitative study has certain limitations. Results reported by participants sampled in four FMUs may not be transferable to other clinical settings with a teaching mission. They should be further validated by conducting other studies in different settings to identify the full range of challenges to consider when implementing AA. Moreover, considering that the AA model is in constant adaptation, without a longitudinal study design, the evolution of the challenges over time cannot be identified. Further studies, investigating the challenges encountered during the change process and the strategies put in place by team members and supervising physicians to overcome them, would be relevant. It is important to mention that the strategies identified and, in some cases, adopted by the actors in this study (e.g., team reconfiguration) have not yet been evaluated. It would be interesting to pursue research and discussion of this issue until a certain level of consensus regarding the solutions will be reached between the various actors involved.

As patients, essential users of the AA model may influence acceptance of this new organizational model and its integration into everyday practice of family physicians and residents in particular, we have explored their perceptions and attitudes regarding implementing AA in an academic unit, in a separate paper.

Finally, evaluation studies would be needed, to test these various strategies to expose future family physicians to best practices during the course of their training; to facilitate the ongoing implementation and adaptation process of this model in their future practice according to the realities of their contexts; and to determine the effects of strategies after they have been adapted to the characteristics of each context and to the particular needs of various professionals.

## Conclusion

Implementing AA in a teaching setting calls for an adaptation of the model according to pedagogical needs. More specifically, the process entails numerous challenges for residents, which require a proactive change approach. Rethinking the training of residents, their role as an active member of the team in implementing AA, and the modalities of their intra- and inter-professional collaboration are strategies to consider, given their potential capacity to anticipate such challenges.

These strategies must be applied in a favorable organizational context, in a primary healthcare organization with an adequate level of available resources, while adopting a dynamic team and collective learning approach.
